# Histogram Analysis of Diffusion Weighted Imaging at 3T is Useful for Prediction of Lymphatic Metastatic Spread, Proliferative Activity, and Cellularity in Thyroid Cancer

**DOI:** 10.3390/ijms18040821

**Published:** 2017-04-12

**Authors:** Stefan Schob, Hans Jonas Meyer, Julia Dieckow, Bhogal Pervinder, Nikolaos Pazaitis, Anne Kathrin Höhn, Nikita Garnov, Diana Horvath-Rizea, Karl-Titus Hoffmann, Alexey Surov

**Affiliations:** 1 Department for Neuroradiology, University Hospital Leipzig, Leipzig 04103, Germany; karl-titus.hoffmann@medizin.uni-leipzig.de; 2 Department for Diagnostic and Interventional Radiology, University Hospital Leipzig, Leipzig 04103, Germany; jonas90.meyer@web.de (H.J.M.); nikita@garnov.de (N.G.); alexey.surov@medizin.uni-leipzig.de (A.S.); 3 Department for Ophthalmology, University Hospital Leipzig, Leipzig 04103, Germany; julia@dieckow.de; 4 Department for Diagnostic and Interventional Neuroradiology, Katharinenhospital Stuttgart, Stuttgart 70174, Germany; bhogalweb@aol.com (B.P.); dihorvath@freenet.de (D.H.-R.); 5 Institute for Pathology, University Hospital Halle-Wittenberg, Martin-Luther-University Halle-Wittenberg, Halle 06112, Germany; nikolaos.pazaitis@uk-halle.de; 6 Institute for Pathology, University Hospital Leipzig, Leipzig 04103, Germany; annekathrin.hoehn@medizin.uni-leipzig.de

**Keywords:** thyroid carcinoma, diffusion weighted imaging, lymphatic metastatic spread, ADC histogram analysis, histopathologic features, Ki-67, p53

## Abstract

Pre-surgical diffusion weighted imaging (DWI) is increasingly important in the context of thyroid cancer for identification of the optimal treatment strategy. It has exemplarily been shown that DWI at 3T can distinguish undifferentiated from well-differentiated thyroid carcinoma, which has decisive implications for the magnitude of surgery. This study used DWI histogram analysis of whole tumor apparent diffusion coefficient (ADC) maps. The primary aim was to discriminate thyroid carcinomas which had already gained the capacity to metastasize lymphatically from those not yet being able to spread via the lymphatic system. The secondary aim was to reflect prognostically important tumor-biological features like cellularity and proliferative activity with ADC histogram analysis. Fifteen patients with follicular-cell derived thyroid cancer were enrolled. Lymph node status, extent of infiltration of surrounding tissue, and Ki-67 and p53 expression were assessed in these patients. DWI was obtained in a 3T system using *b* values of 0, 400, and 800 s/mm^2^. Whole tumor ADC volumes were analyzed using a histogram-based approach. Several ADC parameters showed significant correlations with immunohistopathological parameters. Most importantly, ADC histogram skewness and ADC histogram kurtosis were able to differentiate between nodal negative and nodal positive thyroid carcinoma. Conclusions: histogram analysis of whole ADC tumor volumes has the potential to provide valuable information on tumor biology in thyroid carcinoma. However, further studies are warranted.

## 1. Introduction

The incidence of thyroid cancer, being the most abundant endocrine malignancy, is rapidly increasing [1]. The vast majority of thyroid neoplasms is follicular cell-derived and subsumed under the umbrella categories of papillary thyroid cancer, follicular thyroid cancer, poorly differentiated thyroid cancer, and anaplastic thyroid cancer [2]. Although the overall five-year survival rates of thyroid cancer are 94% in women and 85% in men [3], certain entities of the disease are perpetually associated with poor outcomes (for example the tall cell variant of papillary thyroid cancer and undifferentiated thyroid cancer [1]). Some of the differentiated entities—most of all papillary thyroid cancer variants—frequently metastasize locally via the lymphatic system [4], and resultant local recurrence is not an uncommon scenario [5], leading to significant morbidity.

A variety of therapeutic options is available for thyroid cancer [6], but surgery still remains the predominant treatment [7]. Radical surgery is the most important form of therapy for undifferentiated thyroid cancer [8], and surgical treatment of significant nodal disease in well differentiated thyroid cancer is widely accepted to be associated with improved outcomes in terms of survival and recurrence rates [9]. Nonetheless, extensive surgery in this specific context carries a high risk of therapy-related morbidity like phrenic nerve palsy, brachial plexus palsy, cranial nerve injury, chyle leak, and pneumothorax [10].

Considering the broad spectrum of aggressiveness in thyroid cancer and the resulting necessity for customized treatment, employing presurgical imaging is of great importance, as it allows the thyroid surgeon to identify disease subtypes being associated with increased mortality and morbidity such as metastasizing and undifferentiated thyroid cancer.

Diffusion-weighted magnetic resonance imaging (DWI) has the potential to reveal tumor architectural details like cellular density and proliferative activity in different malignant entities [11,12]. Using a standard echo-planar imaging (EPI) technique, DWI has the capability to differentiate between malignant and benign thyroid nodules [13]. Furthermore, DWI can distinguish manifestations of papillary thyroid cancer with extra-glandular growth from those confined to the thyroid [14]. Using a RESOLVE sequence (which is less prone to susceptibility and motion-induced phase artifacts, has less T2* blurring and provides higher resolution than standard EPI DWI, [15]) in a 3T scanner, DWI even has the capability to distinguish between differentiated and undifferentiated subtypes of thyroid carcinoma [16].

However, in the clinical setting, obtained DWI data is commonly analyzed using a two-dimensional region of interest in the slice of the apparent diffusion coefficient (ADC) map representing the maximum diameter of the tumor. This approach does not account for the regularly encountered heterogeneity of whole tumors and certainly does not reflect the complex micro-architectural properties of malignantly transformed tissue.

An enhanced approach using every voxel of the tumor to compute a histogram of intensity levels could help to further increase prediction of histological features of tumors by magnetic resonance imaging (MRI) [17]. This way, the magnitude of tumor heterogeneity probably is revealed in a fashion superior to the commonly used two-dimensional method [17].

To the best of the authors’ knowledge, only one study used ADC histogram analysis in thyroid cancer to differentiate benign from malignant nodules and furthermore reveal extra-thyroidal growth of papillary thyroid cancer [18]. So far, no studies demonstrated predictability of lymph node involvement by ADC histogram analysis of the primary tumor. Therefore, the primary aim of this study was to investigate the potential of ADC histogram analysis (including percentiles, entropy, skewness, and kurtosis) on data obtained with RESOLVE DWI to distinguish between nodal-negative and nodal-positive thyroid cancer. The discriminability of metastatic from non-metastatic thyroid cancer is of great clinical importance. Hence, this study investigated a promising translational approach that might have the potential to significantly increase the value of clinical-oncological imaging. The secondary aim was to correlate ADC histogram parameters with expression of important prognostic markers like p53 and Ki-67. Last, it aimed to compare our findings with the results of previous studies, which investigated the potential of DWI to predict histopathological features in thyroid cancer.

## 2. Results

### 2.1. Diffusion Weighted Imaging and Immunohistopathology of Thyroid Carcinoma

**Figure 1:**
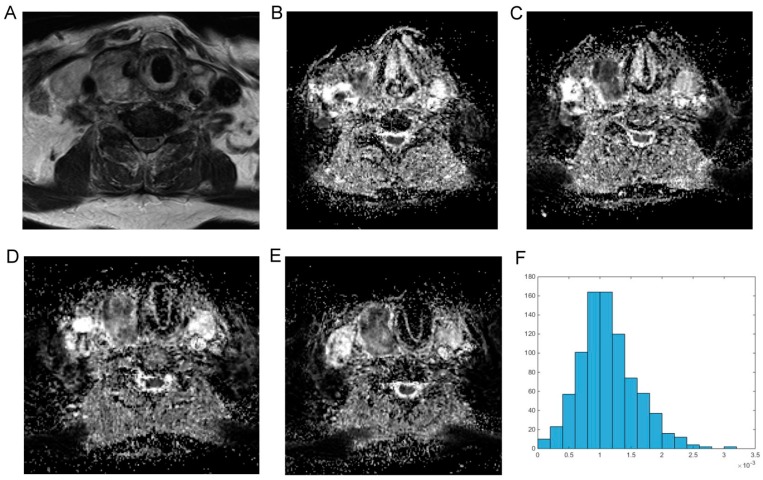
Imaging findings in a patient with follicular thyroid carcinoma. (**A**) Magnetic resonance imaging (T2w axial section) showing a massive inhomogenous enlargement of the right thyroid lobe; (**B**–**E**) represent the apparent diffusion coefficient (ADC) maps of the tumor; (**F**) is the ADC histogram of the whole lesion. The calculated ADC parameters (×10^−5^ mm^2^·s^−1^) are as follows: ADC_min_ = 18.2; ADC_mean_ = 113.3; ADC_max_ = 315.0, mode = 114.4, ADC_median_ = 108.1, P10 = 58.2, P25 = 83.2, P75 = 138.7, and P90 = 176.6. Histogram based parameters are as follows: skewness = 0.59, kurtosis = 3.88, and entropy = 3.21. The *z*-axis in Figure 1F gives the voxel count.

**Figure 2:**
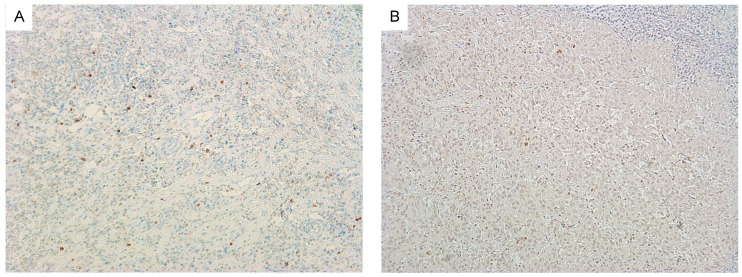
Immunohistochemistry of follicular thyroid carcinoma. (**A**) Shows Ki-67 staining (cell count: 1407, Ki-67 immunoreactiviy: 11%) and (**B**) shows p53 staining (cell count: 1811, p53 immunoreactivity: 36%) of the tumor displayed in Figure 1.

For reasons of clarity and comprehensibility, results of MRI and histopathology were organized in tables. Figure 1 shows MRI findings of a patient with follicular thyroid carcinoma, presenting as heterogeneous enlargement of the right thyroid lobe. The corresponding immunohistological images are shown in Figure 2. The calculated DWI parameters of all investigated thyroid carcinomas are summarized in Table 1 and the corresponding histopathological data is given in Table 2.

### 2.2. Correlation Analysis

**Figure 3:**
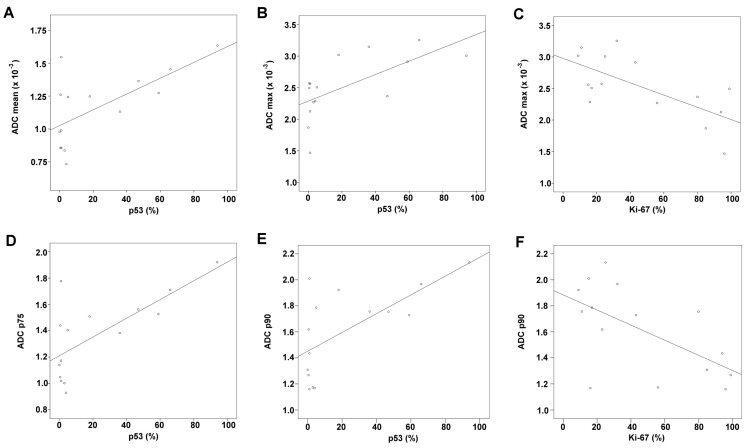

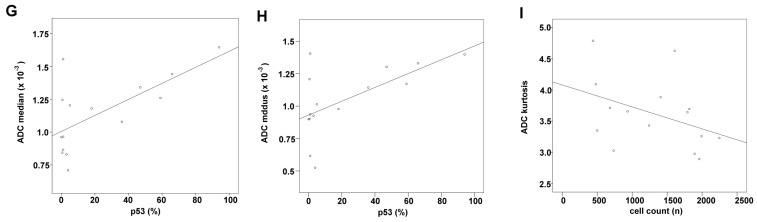
Graphic summary of the significant correlations between imaging and immunohistological findings. *R*^2^-values for the plots shown in Figure 3 are as follows; (**A**) ADC_mean_ & p53: *r*^2^ = 0.438; (**B**) ADC_max_ & p53: *r*^2^ = 0.425; (**C**) ADC_max_ & Ki-67: *r*^2^ = 0.464; (**D**) ADCp75 & p53: *r*^2^ = 0.499; (**E**) ADCp90 & p53: *r*^2^ = 0.431; (**F**) ADCp90 & Ki-67: *r*^2^ = 0.360; (**G**) ADC_median_ & p53: *r*^2^ = 0.440; (**H**) ADC_modus_ & p53: *r*^2^ = 0.377; (**I**) ADC_kurtosis_ & cell count: *r*^2^ = 0.160.

Table 3 displays results of the correlation analysis between immunohistopathological parameters and ADC fractions as well as histogram related parameters. Correlation analysis identified the following, significant correlations: ADC_mean_ with p53 (*r* = 0.548, *p* = 0.034), ADC_max_ with Ki67 (*r* = −0.646, *p* = 0.009) and p53 (*r* = 0.645, *p* = 0.009), ADCp75 with p53 (*r* = 0.537, *p* = 0.025), ADCp90 with Ki67 (*r* = −0.568, *p* = 0.027) and p53 (*r* = 0.588, *p* = 0.021), ADC_median_ with p53 (*r* = 0.556, *p* = 0.032), ADC_modus_ with p53 (*r* = 0.534, *p* = 0.040), and kurtosis with cell count (*r* = −0.571, *p* = 0.026). Figure 3 summarizes the significant correlations graphically and displays them as dot plots.

### 2.3. Group Comparisons

**Figure 4:**
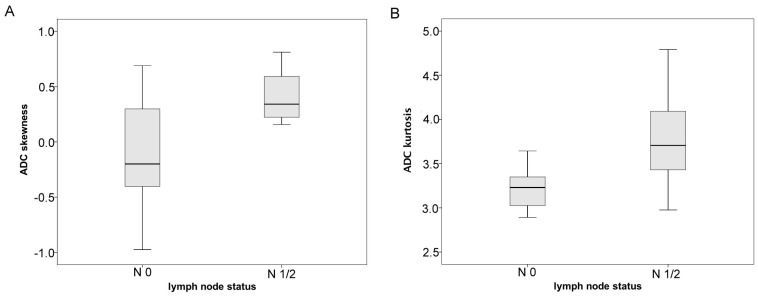
Graphically summarizes the differences in histogram parameters between nodal negative and nodal positive patients with thyroid carcinoma. (**A**) Shows significantly increased ADC histogram skewness in noda-positive compared to nodal-negative patients; (**B**) demonstrates significantly increased values of ADC histogram kurtosis in nodal-positive compared to nodal negative thyroid carcinomas.

Histogram analysis derived ADC values are compared between the nodal negative and the nodal positive group in Figure 4. Levene’s Test revealed homoscedasticity for the nodal-negative and the nodal-positive group only regarding ADC_skewness_ (*p* = 0.015). For all remaining ADC derived histogram parameters, Levene’s Test showed heterogeneity of variance when comparing the nodal-negative and the nodal-positive group. Hence, group comparisons were performed using unpaired *t*-test for ADC_skewness_ and Mann-Whitney-U Test for all remaining parameters. The corresponding *p*-values are given in Table 4. Statistically significant differences were only identified for skewness (*p* = 0.031) and kurtosis (*p* = 0.028). No other significant differences or trends were delineable when comparing thyroid carcinoma patients with restricted vs. advanced infiltration pattern (results not presented).

## 3. Discussion

This study aimed to investigate the potential of 3T RESOLVE DWI using an ADC histogram analysis approach to distinguish between limited and advanced thyroid cancer with reference to the status of lymphatic metastatic dissemination. To the author’s best knowledge, this work is the first to show differences in ADC histogram parameters between nodal-positive and nodal-negative thyroid cancer.

In detail, skewness and kurtosis of the ADC histograms were significantly increased in nodal-positive compared to nodal-negative thyroid cancer. This finding corresponds to previous studies in other malignant tumors, exemplarily clear cell renal cell carcinoma, and rectal cancer, which revealed that increased skewness of ADC histograms is associated with a more advanced disease stage [19,20]. Furthermore, an increase in ADC histogram skewness was observed in patients suffering from recurrent high grade glioma who showed disease progress under anti-proliferative chemotherapy, indicating ongoing proliferation of glioma cells within the tumor [21]. The association between changes in ADC values and altered cellularity in tumors is a well-known phenomenon [22]. Considering this, the findings of the aforementioned studies and our results we hypothesize that the process of lymphatic metastatic spread of thyroid cancer is linked to profound changes in the tissue microarchitecture, related to proliferation of distinct tumor cell clusters and subsequent migration via the lymphatic system, which finds its reflection in corresponding changes of the ADC histogram.

Additionally, this study found significant correlations between ADC histogram analysis derived values of thyroid cancer and corresponding immune-reactivity for p53. p53 has great importance as tumor suppressor and controls cell fate via induction of apoptosis, cell cycle arrest and senescence [23]. Under normal conditions, p53 remains undetectable for its rapid proteasomic degradation [23]. In thyroid cancer, p53 has been used as prognostic marker being associated with favorable outcome [24,25]. ADC mean, ADC max, ADC median, ADC modus, ADC p75 and ADC p90 correlated significantly with p53 expression. In general, increased ADC values of tumors have been shown to be associated with good therapeutic responses [26]. It was thereupon concluded that increased ADC values of thyroid cancer—in consent with previously published work—indicate a favorable prognosis. Furthermore, a clear inverse correlation of ADC max and ADC p90 with Ki-67 expression was identified. Ki-67 is a nuclear protein strictly associated with cell division and widely used in the clinical routine to assess proliferative activity [27].

Increased proliferation of cells, as indicated by increased expression of Ki-67, consecutively decreases the corresponding extracellular space in a given volume of tissue and thereupon reduces water diffusibility, which is reflected by decreased ADC values [22]. Thus—in accordance to other malignancies [11,28,29]—decreased ADC values are associated with an increased proliferation rate within thyroid cancer tissue.

This study furthermore identified a significant inverse correlation between cell count and kurtosis. Only few studies investigated the potential of ADC kurtosis to reflect histological properties, for example Chandarana and colleagues were able to differentiate clear cell from papillary subtype of renal cell cancer by means of ADC kurtosis [30]. It is therefore concluded that ADC histogram kurtosis provides additional insight in tumor-architectural details, but further studies are necessary to validate this finding in order to further elaborate the significance of this parameter. Conventionally, ADC_mean_ and ADC_min_ were used to investigate histopathological features like cellularity of tumors in vivo [22]. However, classical ADC parameters like ADC_mean_ and ADC_min_ are strongly scanner-dependent and cannot be used to compare patients investigated in different MRI devices without normalization. In contrary, histogram parameters estimate characteristics of the ADC distribution, which is not scanner-dependent like the absolute ADC values. Therefore, ADC derived histogram parameters (skewness, entropy, kurtosis) might be superior when investigating histopathological features in vivo using more than one MRI scanner in a singular study.

This study suffers from few limitations. The major limitation is the small number of patients included in this study. Furthermore, this study did not include all clinically relevant subtypes of thyroid cancer, exemplarily medullary thyroid carcinomas were not investigated. Therefore, future works including greater cohorts with different histopathological subtypes have to confirm these findings and further elucidate the relationship between histopathological findings and ADC alterations. Also, ADC histogram analysis was performed by a single, experienced reader. The suitability of histogram analysis for the clinical routine necessitates assessment of inter-reader and intra-reader variability including readers with different levels of experience. A future work needs to investigate these phenomena in a larger cohort.

ADC histogram analysis can provide more detailed information on diffusion characteristics of tumors than commonly obtained ADC parameters. For example, a previously published study demonstrated that common ADC parameters (mean, max, and min) did not reflect histopathological features like cellularity and proliferative activity in thyroid carcinoma [16]. In contrast, this study demonstrated that certain ADC histogram parameters reflect distinct histopathological features very well. Although it has proven to be a very sensitive tool for detection of microstructural changes, the specificity of ADC histogram parameters for the underlying histological changes is unclear. Characteristic changes of ADC histogram parameters in different tumor entities might be related to very different histological changes. Therefore, the significance of ADC histogram analysis should be investigated in a tumor-specific manner.

## 4. Materials and Methods

This retrospective study was approved (No. 2014-99) by the local research ethics committee of the Martin-Luther-University Halle-Wittenberg.

### 4.1. Patients

The radiological database for thyroid carcinoma was reviewed. In total, 20 patients were identified, but only 15 patients with histopathologically confirmed thyroid carcinoma had received proper DWI (using the RESOLVE sequence) and were therefore enrolled in our study.

The patient group was comprised of one male and 14 female patients. The mean age was 67 years (with a standard deviation of 12.9 years). The distribution of histopathological subtypes was as follows; follicular thyroid carcinoma: *n* = 4, papillary thyroid carcinoma: *n* = 5, anaplastic thyroid carcinoma: *n* = 6. Five patients were diagnosed with nodal negative thyroid cancer, and 10 patients had pathologically confirmed lymph node metastases. One patient was diagnosed with distant metastatic disease (pulmonary and pleural manifestation). Infiltration pattern ranged from restriction to the thyroid gland to advanced infiltration including infiltration of the trachea, esophagus, and internal jugular vein. An overview of demographic, clinical and pathological information is given in Table 5.

### 4.2. MRI

MRI of the neck was performed for all patients using a 3T device (Magnetom Skyra, Siemens, Erlangen, Germany). The imaging protocol included the following sequences:

1. axial T2 weighted (T2w) turbo spin echo (TSE) sequence (TR/TE: 4000/69, flip angle: 150°, slice thickness: 4 mm, acquisition matrix: 200 × 222, field of view: 100 mm);
2. axial T1 weighted (T1w) turbo spin echo (TSE ) sequences (TR/TE: 765/9.5, flip angle: 150°, slice thickness: 5 mm, acquisition matrix: 200 × 222, field of view: 100 mm) before and after intravenous application of contrast medium (gadopentate dimeglumine, Magnevist^®^, Bayer Schering Pharma, Leverkusen, Germany);
3. axial DWI (readout-segmented, multi-shot EPI sequence; TR/TE: 5400/69, flip angle 180°, slice thickness: 4 mm, acquisition matrix: 200 × 222, field of view: 100 mm) with b values of 0, 400 and 800 s/mm^2^. ADC maps were generated automatically by the implemented software package and analyzed as described previously [28].

All images were available in digital form and were analyzed by an experienced radiologist without knowledge of the histopathological diagnosis on a PACS workstation (Centricity PACS, GE Medical Systems, Milwaukee, WI, USA). Figure 1 shows a representative axial T2 weighted image of follicular thyroid carcinoma and corresponding axial ADC images of the whole tumor, which were used for histogram analysis (also displayed in Figure 1).

### 4.3. Histogram Analysis of ADC Values

DWI data was transferred in DICOM format and processed offline with a custom-made Matlab-based application (The Mathworks, Natick, MA, USA) on a standard windows operated system. The ADC maps were displayed within a graphical user interface (GUI) that enables the reader to scroll through the slices and draw a volume of interest (VOI) at the tumor’s boundary. The VOI was created by manually drawing regions of interest (ROIs) along the margin of the tumor using all slices displaying the tumor (whole lesion measure). All measures were performed by one author (AS). The ROIs were modified in the GUI and saved (in Matlab-specific format) for later processing. After setting the ROIs, the following parameters were calculated and given in a spreadsheet format: ROI volume (cm^3^), mean (ADC_mean_), maximum (ADC_max_), minimum (ADC_min_), median (ADC_median_), modus (ADC_modus_), and the following percentils: 10th (ADCp10), 25th (ADCp25), 75th (ADCp75), and 90th (ADCp90). Additionally, histogram-based characteristics of the VOI—kurtosis, skewness, and entropy—were computed. All calculations were performed using in-build Matlab functions.

### 4.4. Histopathology and Immunohistochemistry

All thyroid carcinomas were surgically resected and histopathologically analysed. In every case, the proliferation index was estimated on Ki-67 antigen stained specimens using MIB-1 monoclonal antibody (DakoCytomation, Glostrup, Denmark) as reported previously [31]. Furthermore, p53 index was estimated using monoclonal antibody p57, clone DO-7 (DakoCytomation). Two high power fields (0.16 mm^2^ per field, ×400) were analysed. The area with the highest number of positive nuclei was selected. Figure 2 exemplarily shows Ki-67 and p53 immunostaining of a follicular thyroid carcinoma. Additionally, cellular density was calculated for each tumor as average cell count per five high power fields (×400). Furthermore, average nuclear area and total nuclear area were estimated using ImageJ package 1.48v (National Institute of Health, Bethesda, MD, USA) as described previously [11]. All histopathological sections were analysed using a research microscope Jenalumar equipped with a Diagnostic instruments camera 4.2 (Zeiss, Jena, Germany).

### 4.5. Statistical Analysis

Statistical analysis was performed using IBM SPSS 23™ (SPSS Inc., Chicago, IL, USA). Collected data was first evaluated by means of descriptive statistics. Correlative analysis was then performed using Spearman’s correlation coefficient in order to analyze associations between histogram analysis derived values of ADC and (immuno-) histopathological parameters. Subsequently, Levene’s Test for homogeneity of variance was performed to assess the equality of variances of ADC derived histogram parameters between different groups of thyroid carcinoma patients in order to identify the suitable test for group comparisons. In case of homoscedasticity, unpaired t test was performed to compare values among different (e.g., the metastatic and the non-metastatic) groups. In case of heteroscedasticity, Mann-Whitney-*U* test was performed to compare values among the different groups. Group comparisons were performed for nodal negative vs. nodal positive patients and patients with restricted (thyroid gland and trachea) vs. advanced (trachea, esophagus, jugular vein) infiltration pattern. Since only one patient with distant metastatic disease was included, a sufficient group comparison between M0 and M1 patients could not be performed. *p*-Values ≤ 0.05 were considered as statistically significant.

## 5. Conclusions

This exploratory study revealed significant differences in ADC histogram skewness and kurtosis comparing nodal negative and nodal positive thyroid cancer. Significant correlations between different ADC parameters were identified with p53, Ki-67, and cell count, substantiating the potential of ADC as an important prognostic imaging biomarker. This information certainly has the potential to aid thyroid surgeons in identifying the optimal treatment strategy for patients with thyroid cancer. Further studies investigating a greater cohort of patients are necessary to confirm these findings.

## Figures and Tables

**Table 1:** Diffusion weighted imaging and related histogram parameters of thyroid carcinoma based on *n* = 15 patients.

DWI Related Parameters	Median	Range	Minimum–Maximum
ADC_mean_, ×10^−5^ mm^2^·s^−1^	124.30	90	73–163
ADC_min_, ×10^−5^ mm^2^·s^−1^	14.90	53	0.2–53
ADC_max_, ×10^−5^ mm^2^·s^−1^	250.70	179	147–325
P10 ADC, ×10^−5^ mm^2^·s^−1^	72.10	85	30–114
P25 ADC, ×10^−5^ mm^2^·s^−1^	91.90	84	52–136
P75 ADC, ×10^−5^ mm^2^·s^−1^	140.40	99	93–192
P90 ADC, ×10^−5^ mm^2^·s^−1^	172.82	116	97–213
Median ADC, ×10^−5^ mm^2^·s^−1^	118.00	94	71–165
Mode ADC, ×10^−5^ mm^2^·s^−1^	101.40	88	53–141
Kurtosis	3.64	1.90	2.89–4.79
Skewness	0.30	1.79	−0.97–0.81
Entropy	3.27	1.98	2.75–4.72

**Table 2:** Estimated immunohistopathological parameters of thyroid carcinoma (*n* = 15).

Parameters	Median	Range	Minimum–Maximum
Cell count, *n*	1407	1808	439–2247
Ki 67, %	32.0	90	9–99
p53, %	4.0	94	0–94
Total nuclear area, µm^2^	71,735	148,620	14,649–163,269
Average nuclear area, µm^2^	53.0	61	33–94

**Table 3:** Results of Spearman’s rank order correlation analysis between DWI and immunohistological parameters (*n* = 15).

ADC Parameters and Histogram Values	Cell Count	p53	Ki-67	Total Nuclear Area	Average Nuclear Area
ADC_mean_, ×10^−3^ mm^2^·s^−1^	*r* = 0.429	*r* = 0.548	*r* = −0.325	*r* = 0.389	*r* = 0.034
	*p* = 0.111	*p* = 0.034	*p* = 0.237	*p* = 0.152	*p* = 0.904
ADC_min_, ×10^−3^ mm^2^·s^−1^	*r* = 0.256	*r* = 0.244	*r* = −0.241	*r* = 0.163	*r* = −0.208
	*p* = 0.358	*p* = 0.381	*p* =0.386	*p* = 0.562	*p* = 0.456
ADC_max_, ×10^−3^ mm^2^ s^−1^	*r* = 0.372	*r* = 0.645	*r* = −0.646	*r = 0.461*	*r* = 0.155
	*p* = 0.173	*p* = 0.009	*p* = 0.009	*p = 0.084*	*p* = 0.580
ADC p10, ×10^−3^ mm^2^·s^−1^	*r* = 0.361	*r* = 0.409	*r* = 0.289	*r* = 0.275	*r* = −0.079
	*p* = 0.187	*p* =0.130	*p* = 0.296	*p* = 0.321	*p* = 0.781
ADC p25, ×10^−3^ mm^2^·s^−1^	*r* = 0.375	*r = 0.509*	*r* = 0.361	*r* = 0.311	*r* = −0.064
	*p* = 0.168	*p = 0.053*	*p* = 0.187	*p* = 0.260	*p* = 0.820
ADC p75, ×10^−3^ mm^2^·s^−1^	*r =* 0.450	*r* = 0.537	*r* = −0.343	*r* = 0.411	*r* = 0.055
	*p* = 0.092	*p* = 0.025	*p* =0.211	*p* =0.128	*p* = 0.845
ADC p90, ×10^−3^ mm^2^·s^−1^	*r* = 0.289	*r* = 0.588	*r* = −0.568	*r* = 0.300	*r* = 0.075
	*p* = 0.296	*p* = 0.021	*p* = 0.027	*p* = 0.277	*p* = 0.790
Median ADC, ×10^−3^ mm^2^·s^−1^	*r* = 0.414	*r* = 0.556	*r* = −0.314	*r* = 0.361	*r* = −0.020
	*p* = 0.125	*p* = 0.032	*p* = 0.254	*p* = 0.187	*p* = 0.945
Mode ADC, ×10^−3^ mm^2^·s^−1^	*r* = 0.496	*r* = 0.534	*r* = −0.357	*r* = 0.432	*r* = −0.149
	*p* = 0.060	*p* = 0.040	*p* = 0.191	*p* = 0.108	*p* = 0.682
Kurtosis	*r* = −0.571	*r* = −0.262	*r* = −0.314	*r* = −0.411	*r* = −0.182
	*p* = 0.026	*p* =0.346	*p* = 0.254	*p* = 0.128	*p* = 0.516
Skewness	*r* = −0.229	*r* = −0.004	*r* = −0.389	*r* = 0.011	*r* = 0.186
	*p* = 0.413	*p* = 0.990	*p* = 0.152	*p* = 0.970	*p* = 0.507
Entropy	*r* = 0.243	*r* = −0.240	*r* = 0.289	*r* = 0.225	*r* = 0.316
	*p* = 0.383	*p* = 0.389	*p* = 0.296	*p* = 0.420	*p* = 0.251

**Table 4:** Group comparison of ADC and histogram parameters of thyroid carcinomas with (N1/2, *n* = 10 patients) and without lymphatic metastatic dissemination (N0, *n* = 5 patients).

ADC Parameters and Histogram Values	N0 Mean ± SD		N1/2 Mean ± SD		Group Comparison: *p*-Values
ADC_mean_, ×10^−5^ mm^2^·s^−1^	125.25	34.1	111.41	25.00	0.513
ADC_min_, ×10^−5^ mm^2^·s^−1^	28.26	17.30	14.02	16.90	0.075
ADC_max_, ×10^−5^ mm^2^·s^−1^	238.44	69.40	259.43	38.50	0.768
P10 ADC, ×10^−5^ mm^2^·s^−1^	82.15	26.17	69.14	23.50	0.371
P25 ADC, ×10^−5^ mm^2^·s^−1^	102.25	30.00	89.19	23.30	0.440
P75 ADC, ×10^−5^ mm^2^·s^−1^	147.26	39.14	131.75	26.43	0.440
P90 ADC, ×10^−5^ mm^2^·s^−1^	170.69	44.15	156.55	28.50	0.440
Median ADC, ×10^−5^ mm^2^·s^−1^	124.14	34.86	109.19	25.50	0.513
Mode ADC, ×10^−5^ mm^2^·s^−1^	112.32	25.56	101.39	27.50	0.594
Kurtosis	3.23	0.29	3.81	0.57	*0.028*
Skewness	−0.12	0.64	0.41	0.21	0.031
Entropy	3.56	0.66	3.5	0.71	0.768

**Table 5:** Demographic and pathological data of the investigated thyroid carcinoma patients.

Case	Age	Gender	Histological Subtype	Infiltration Pattern	M Stage	N Stage
1	91	female	anaplastic	trachea	0	1
2	60	female	papillary	trachea	0	1
3	73	male	papillary	trachea, esophagus	0	1
4	68	female	papillary	trachea, esophagus internal jugular vein	0	0
5	73	female	papillary	trachea	0	1
6	67	female	anaplastic	Trachea internal jugular vein	1	2
7	73	female	anaplastic	trachea, esophagus	0	0
8	41	female	follicular	trachea	0	1
9	72	female	anaplastic	none	0	1
10	59	female	anaplastic	trachea	0	1
11	83	female	papillary	trachea	0	0
12	77	female	follicular	trachea	0	1
13	52	female	anaplastic	trachea	0	0
14	51	female	follicular	trachea	0	0
15	66	female	anaplastic	trachea	0	1

## Abbreviations

MDPI	Multidisciplinary Digital Publishing Institute
DOAJ	Directory of Open Access Journals
TLA	Three Letter Acronym
LD	linear Dichroism

## References

[1] Katoh H., Yamashita K., Enomoto T., Watanabe M. (2015). Classification and general considerations of thyroid cancer. Ann. Clin. Pathol..

[2] Dralle H., Machens A., Basa J., Fatourechi V., Franceschi S., Hay I.D., Nikiforov Y.E., Pacini F., Pasieka J.L., Sherman S.I. (2015). Follicular cell-derived thyroid cancer. Nat. Rev. Dis. Prim..

[3] Paschke R., Lincke T., Müller S.P., Kreissl M.C., Dralle H., Fassnacht M. (2015). The treatment of well-differentiated thyroid carcinoma. Dtsch. Arztebl. Int..

[4] Nixon I.J., Shaha A.R. (2013). Management of regional nodes in thyroid cancer. Oral Oncol..

[5] Shaha A.R. (2012). Recurrent differentiated thyroid cancer. Endocr. Pract..

[6] Ferrari S.M., Fallahi P., Politti U., Materazzi G., Baldini E., Ulisse S., Miccoli P., Antonelli A. (2015). Molecular targeted therapies of aggressive thyroid cancer. Front Endocrinol..

[7] Cabanillas M.E., Dadu R., Hu M.I., Lu C., Gunn G.B., Grubbs E.G., Lai S.Y., Williams M.D. (2015). Thyroid gland malignancies. Hematol. Oncol. Clin. N. Am..

[8] Wendler J., Kroiss M., Gast K., Kreissl M.C., Allelein S., Lichtenauer U., Blaser R., Spitzweg C., Fassnacht M., Schott M. (2016). Clinical presentation, treatment and outcome of anaplastic thyroid carcinoma: Results of a multicenter study in Germany. Eur. J. Endocrinol..

[9] Asimakopoulos P., Nixon I.J., Shaha A.R. (2017). Differentiated and medullary thyroid cancer: Surgical management of cervical lymph nodes. Clin. Oncol..

[10] Mizrachi A., Shaha A.R. (2016). Lymph node dissection for differentiated thyroid cancer. Mol. Imaging Radionucl. Ther..

[11] Schob S., Meyer J., Gawlitza M., Frydrychowicz C., Müller W., Preuss M., Bure L., Quäschling U., Hoffmann K.-T., Surov A. (2016). Diffusion-weighted MRI reflects proliferative activity in primary CNS lymphoma. PLoS ONE.

[12] Surov A., Stumpp P., Meyer H.J., Gawlitza M., Höhn A.-K., Boehm A., Sabri O., Kahn T., Purz S. (2016). Simultaneous ^18^F-FDG-PET/MRI: Associations between diffusion, glucose metabolism and histopathological parameters in patients with head and neck squamous cell carcinoma. Oral Oncol..

[13] Khizer A.T., Raza S., Slehria A.-U.-R. (2015). Diffusion-weighted MR imaging and ADC mapping in differentiating benign from malignant thyroid nodules. J. Coll. Physicians Surg. Pak..

[14] Lu Y., Moreira A.L., Hatzoglou V., Stambuk H.E., Gonen M., Mazaheri Y., Deasy J.O., Shaha A.R., Tuttle R.M., Shukla-Dave A. (2015). Using diffusion-weighted MRI to predict aggressive histological features in papillary thyroid carcinoma: A novel tool for pre-operative risk stratification in thyroid cancer. Thyroid.

[15] Porter D.A., Heidemann R.M. (2009). High resolution diffusion-weighted imaging using readout-segmented echo-planar imaging, parallel imaging and a two-dimensional navigator-based reacquisition. Magn. Reson. Med..

[16] Schob S., Voigt P., Bure L., Meyer H.J., Wickenhauser C., Behrmann C., Höhn A., Kachel P., Dralle H., Hoffmann K.-T., Surov A. (2016). Diffusion-weighted imaging using a readout-segmented, multishot EPI sequence at 3T distinguishes between morphologically differentiated and undifferentiated subtypes of thyroid carcinoma—A preliminary study. Transl. Oncol..

[17] Just N. (2014). Improving tumour heterogeneity MRI assessment with histograms. Br. J. Cancer.

[18] Hao Y., Pan C., Chen W., Li T., Zhu W., Qi J. (2016). Differentiation between malignant and benign thyroid nodules and stratification of papillary thyroid cancer with aggressive histological features: Whole-lesion diffusion-weighted imaging histogram analysis. J. Magn. Reson. Imaging.

[19] Kierans A.S., Rusinek H., Lee A., Shaikh M.B., Triolo M., Huang W.C., Chandarana H. (2014). Textural differences in apparent diffusion coefficient between low- and high-stage clear cell renal cell carcinoma. Am. J. Roentgenol..

[20] Liu L., Liu Y., Xu L., Li Z., Lv H., Dong N., Li W., Yang Z., Wang Z., Jin E. (2016). Application of texture analysis based on apparent diffusion coefficient maps in discriminating different stages of rectal cancer. J. Magn. Reson. Imaging.

[21] Nowosielski M., Recheis W., Goebel G., Güler O., Tinkhauser G., Kostron H., Schocke M., Gotwald T., Stockhammer G., Hutterer M. (2011). ADC histograms predict response to anti-angiogenic therapy in patients with recurrent high-grade glioma. Neuroradiology.

[22] Chen L., Liu M., Bao J., Xia Y., Zhang J., Zhang L., Huang X., Wang J. (2013). The correlation between apparent diffusion coefficient and tumor cellularity in patients: A meta-analysis. PLoS ONE.

[23] Wang Z., Sun Y. (2010). Targeting p53 for novel anticancer therapy. Transl. Oncol..

[24] Godballe C., Asschenfeldt P., Jørgensen K.E., Bastholt L., Clausen P.P., Hansen T.P., Hansen O., Bentzen S.M. (1998). Prognostic factors in papillary and follicular thyroid carcinomas: P53 expression is a significant indicator of prognosis. Laryngoscope.

[25] Bachmann K., Pawliska D., Kaifi J., Schurr P., Zörb J., Mann O., Kahl H.J., Izbicki J.R., Strate T. (2006). P53 is an independent prognostic factor for survival in thyroid cancer. Anticancer Res..

[26] Padhani A.R., Liu G., Mu-Koh D., Chenevert T.L., Thoeny H.C., Takahara T., Dzik-Jurasz A., Ross B.D., Van Cauteren M., Collins D. (2009). Diffusion-weighted magnetic resonance imaging as a cancer biomarker: Consensus and recommendations. Neoplasia.

[27] Schlüter C., Duchrow M., Wohlenberg C. (1993). The cell proliferation-associated antigen of antibody Ki-67: A very large, ubiquitous nuclear protein with numerous repeated elements, representing a new kind of cell cycle-maintaining proteins. J. Cell Biol..

[28] Surov A., Caysa H., Wienke A., Spielmann R.P., Fiedler E. (2015). Correlation between different ADC fractions, cell count, Ki-67, total nucleic areas and average nucleic areas in meningothelial meningiomas. Anticancer Res..

[29] Chen L., Zhang J., Chen Y., Wang W., Zhou X., Yan X., Wang J. (2014). Relationship between apparent diffusion coefficient and tumour cellularity in lung cancer. PLoS ONE.

[30] Chandarana H., Rosenkrantz A.B., Mussi T.C., Kim S., Ahmad A.A., Raj S.D., McMenamy J., Melamed J., Babb J.S., Kiefer B. (2012). Histogram analysis of whole-lesion enhancement in differentiating clear cell from papillary subtype of renal cell cancer. Radiology.

[31] Surov A., Gottschling S., Mawrin C., Prell J., Spielmann R.P., Wienke A., Fiedler E. (2015). Diffusion-weighted imaging in meningioma: Prediction of tumor grade and association with histopathological parameters. Transl. Oncol..

